# Integrated Bioinformatic and Experimental Analysis of PTEN and DNMT1 Regulation in NSCLC

**DOI:** 10.3390/biomedicines14081813

**Published:** 2026-08-12

**Authors:** Muhamed A. El Nobey, Abdulkader M. Shaikh Omar, Ashwaq H. Batawi, Amani Alharthi, Eman Hillal Althubaiti, Maha Ali Alghamdi, Sarah A. Altalhi, Tahani Bakhsh, Zainab M. Al Aamri

**Affiliations:** 1 Department of Biological Sciences, Faculty of Science, King Abdulaziz University, P.O. Box 80203, Jeddah 21589, Saudi Arabia; 2 Princess Doctor Najla Bint Saud Al Saud Distinguished Research Centre for Biotechnology, King Abdulaziz University, Jeddah 21589, Saudi Arabia; 3 Department of Biology, College of Science in Zulfi, Majmaah University, Majmaah 11952, Saudi Arabia; 4 Department of Biotechnology, College of Science, Taif University, Taif 21944, Saudi Arabia; 5 Department of Biological Science, College of Science, University of Jeddah, P.O. Box 80327, Jeddah 21589, Saudi Arabia; 6 Department of Biology, College of Sciences, Umm Al-Qura University, Makkah 21955, Saudi Arabia

**Keywords:** PTEN, DNMT1, 5-aza-dC, DNA methylation, miR-148a-3p, LUSC, LUAD

## Abstract

**Background/Objectives:** Loss of phosphatase and tensin homolog (*PTEN*) activity is a frequent feature of non-small-cell lung cancer (NSCLC), and epigenetic repression may contribute to its reduced expression. This study investigated the effects of 5-aza-2′-deoxycytidine (5-aza-dC) on PTEN, DNA methyltransferase 1 (DNMT1), *PTEN* promoter methylation-specific amplification patterns, and miR-148a-3p expression in NSCLC models. **Methods:** Publicly available cancer-genomics datasets were analyzed to compare *PTEN* and *DNMT1* transcript abundance and to assess the association between *PTEN* methylation and transcript abundance in lung adenocarcinoma (LUAD) and lung squamous cell carcinoma (LUSC). A549 and H460 cells were exposed to 2.5 or 5 µM 5-aza-dC for 72 h. Reverse-transcription quantitative PCR (RT-qPCR), Western blotting, methylation-specific PCR (MSP-PCR), and 3-(4,5-dimethylthiazol-2-yl)-2,5-diphenyltetrazolium bromide (MTT) assays were used to evaluate RNA expression, protein abundance, methylation-specific amplification, and metabolic activity, respectively. **Results:** Bioinformatic analyses showed lower *PTEN* and higher *DNMT1* expression in both NSCLC subtypes, together with inverse associations between *PTEN* methylation and transcript abundance. In both cell lines, 5-aza-dC reduced MTT metabolic activity and DNMT1 expression. PTEN mRNA and protein abundance increased significantly at 5 µM, whereas no significant changes were detected at 2.5 µM. MSP-PCR revealed persistent heterogeneous PTEN methylation-specific amplification patterns without clear evidence of progressive promoter demethylation. miR-148a-3p exhibited a biphasic response in A549 cells but remained unchanged in H460 cells. **Conclusions:** These findings support an association between 5-aza-dC exposure, increased *PTEN* expression, and reduced *DNMT1* expression in NSCLC cells. Quantitative methylation analysis and mechanistic validation are required to clarify the contribution of miR-148a-3p to this regulatory association.

## 1. Introduction

Lung cancer continues to account for a large proportion of cancer deaths, and most cases are classified as non-small-cell lung cancer (NSCLC) [1,2,3]. Although molecularly targeted agents, immunotherapies, and improved treatment strategies have expanded the available therapeutic options, outcomes for patients with advanced disease remain unsatisfactory [4]. Alongside genomic alterations, NSCLC biology is shaped by epigenetic processes that change transcriptional activity without modifying the DNA sequence. One such process is promoter hypermethylation, which can stably reduce tumor-suppressor gene expression and thereby facilitate tumor growth and therapeutic resistance [5].

Phosphatase and tensin homolog (PTEN), a tumor suppressor encoded on chromosome 10, restrains phosphoinositide 3-kinase/protein kinase B (PI3K/AKT) signaling and thereby limits proliferative, survival, and metabolic signals [6]. Its function may be diminished in lung cancer through deletion, mutation, transcriptional suppression, post-transcriptional regulation, or promoter methylation [7,8]. Maintenance of genomic methylation patterns depends largely on DNA methyltransferase 1 (DNMT1), which preferentially methylates newly synthesized DNA at previously methylated sites [9,10]. Persistent DNMT1 activity can consequently stabilize the repression of tumor-suppressor loci in malignant cells.

miR-148a-3p was selected as a candidate regulatory microRNA (miRNA) because of its reported involvement in *DNMT1* regulation and methylation-dependent tumor-suppressor gene reactivation [11,12,13,14]. Network analysis further linked miR-148a-3p to *DNMT1*- and *PTEN*-related pathways; however, its response to *DNMT* inhibition and association with PTEN re-expression in NSCLC remain unclear.

5-aza-2′-deoxycytidine (5-aza-dC) is incorporated into DNA during replication and traps DNA methyltransferases in covalent complexes, leading to depletion of active DNMT1 and gradual loss of methylation during cell division [15]. However, *PTEN* responses to 5-aza-dC may vary among NSCLC cell backgrounds, and it is uncertain whether any change in *PTEN* expression occurs in parallel with altered miR-148a-3p expression.

To address these questions, we integrated analyses of public NSCLC datasets with experiments in A549 and H460 cells. The computational component compared *PTEN* and *DNMT1* expression in tumor and non-cancerous lung tissues and examined the relationship between *PTEN* methylation and transcript abundance. The cell-based component evaluated the effects of 5-aza-dC on DNMT1, PTEN, *PTEN* methylation-specific amplification, and miR-148a-3p.

## 2. Materials and Methods

### 2.1. Bioinformatics Analysis

Publicly available cancer genomics platforms were used to examine *PTEN* and DNMT1 expression, *PTEN* promoter methylation, and miR-148a-3p-associated gene interactions in NSCLC. Gene Expression Profiling Interactive Analysis 2 (GEPIA2) (http://gepia2.cancer-pku.cn/ (accessed on 1 June 2026)) was used to compare *PTEN* and *DNMT1* transcript abundance between LUAD and LUSC tumors and corresponding normal lung tissues from The Cancer Genome Atlas (TCGA) and the Genotype-Tissue Expression (GTEx) datasets. Identical dataset and analysis settings were applied to both genes [16], and statistical significance was defined as *p* < 0.05. *PTEN* promoter methylation was compared between tumor and normal tissues using UALCAN (https://ualcan.path.uab.edu/ (accessed on 1 June 2026)) [17], whereas the cBioPortal cancer genomics platform (https://www.cbioportal.org/ (accessed on 1 June 2026)) was used to assess the association between *PTEN* methylation and mRNA expression in LUAD and LUSC cohorts [18]. Potential associations of miR-148a-3p with *PTEN*, *DNMT1*, and other genes were explored using the microRNA-centric network analysis platform (miRNet) (https://www.mirnet.ca/ (accessed on 1 June 2026)) [19]. The resulting interaction network was exported and visualized using Cytoscape (version 3.10.4). GEPIA2, UALCAN, cBioPortal, and miRNet were all accessed on 15 November 2025.

### 2.2. Cell Culture

A549 and H460 cells were used as adenocarcinoma and large-cell NSCLC models, respectively, to assess 5-aza-dC responses across distinct histological and molecular backgrounds. No LUSC cell line was included. Both cell lines were obtained from the American Type Culture Collection (ATCC, Manassas, VA, USA) and maintained in Roswell Park Memorial Institute 1640 (Gibco, Thermo Fisher Scientific, Waltham, MA, USA) medium supplemented with 10% fetal bovine serum (FBS; Gibco, Thermo Fisher Scientific, Waltham, MA, USA), 100 U/mL penicillin (Sigma-Aldrich, St. Louis, MO, USA), and 100 µg/mL streptomycin (Lonza Walkersville, Inc., Walkersville, MD, USA). Cells were incubated at 37 °C in a humidified atmosphere containing 5% CO_2_.

### 2.3. Preparation and Administration of 5-aza-dC

5-aza-2′-deoxycytidine (Santa Cruz Biotechnology, Dallas, TX, USA; cat. no. sc-202424) was dissolved in dimethyl sulfoxide to prepare a stock solution. The stock was divided into single-use aliquots and maintained at −80 °C to minimize repeated freezing and thawing. Fresh working dilutions were prepared in complete culture medium immediately before each treatment. For molecular assays, A549 and H460 cells were plated at a density of 1 × 10^5^ cells in 25 cm^2^ culture flasks and incubated overnight to allow cell attachment. The concentrations of 2.5 and 5 µM were selected as experimental concentrations for evaluating the molecular effects of 5-aza-dC in A549 and H460 cells. The cultures were then exposed to 5-aza-dC at concentrations of 2.5 and 5 µM for 72 h. Drug-containing medium was replaced every 24 h. Vehicle-control cultures received 0.1% dimethyl sulfoxide (DMSO) and underwent the same medium-replacement schedule.

### 2.4. Assessment of Cellular Metabolic Activity

Cellular metabolic activity following 5-aza-dC exposure was evaluated using the 3-(4,5-dimethylthiazol-2-yl)-2,5-diphenyltetrazolium bromide (MTT; Sigma-Aldrich, St. Louis, MO, USA) assay. A549 and H460 cells were seeded in 96-well plates at a density of 5 × 10^3^ cells per well in 100 µL of complete culture medium and incubated for 24 h to allow attachment. Cells were subsequently treated with 5-aza-dC at concentrations of 0.03, 0.1, 0.3, 1, 3, 10, 30, 100, and 300 µM for 72 h. Drug-containing medium was replaced every 24 h throughout the 72-h exposure period. Vehicle-control cells received 0.1% dimethyl sulfoxide (DMSO; Sigma-Aldrich, St. Louis, MO, USA) and underwent the same medium-replacement schedule. At the end of the treatment period, the culture medium was removed, and 20 µL of MTT solution prepared at 1 mg/mL was added to each well containing 100 µL of phosphate-buffered saline. After incubation for 4 h at 37 °C, the resulting formazan crystals were dissolved in 100 µL of absolute DMSO. Absorbance was measured at 570 nm using a FLUOstar Omega multi-well plate reader (BMG LABTECH, Ortenberg, Germany). Metabolic activity was calculated relative to the vehicle-treated control. Three independent biological experiments were performed, with three technical replicate wells included for each concentration in every experiment. Concentration–response curves were fitted using a four-parameter logistic nonlinear regression model in GraphPad Prism version 9.5 (GraphPad Software, San Diego, CA, USA), and the half-maximal inhibitory concentration (IC_50_) was calculated for each cell line.

### 2.5. RNA Isolation and RT-qPCR Workflow

Total RNA was isolated from vehicle-treated and 5-aza-dC-treated cells using the RNeasy Mini Kit (QIAGEN GmbH, Hilden, Germany; cat. no. 74104). RNA concentration and purity were assessed spectrophotometrically. For *PTEN* and *DNMT1* analysis, cDNA was synthesized from 1 µg of total RNA using the QuantiTect Reverse Transcription Kit (QIAGEN GmbH, Hilden, Germany). For miR-148a-3p analysis, cDNA was synthesized from total RNA using a miR-148a-3p-specific stem-loop reverse-transcription primer according to the established protocol. Gene-specific quantitative PCR was performed using PowerUp SYBR Green Master Mix (Applied Biosystems, Thermo Fisher Scientific, Waltham, MA, USA) on a CFX Opus 96 Real-Time PCR System (Bio-Rad Laboratories, Inc., Hercules, CA, USA), with the primer sequences provided in Table 1. *PTEN* and *DNMT1* expression levels were normalized to *GAPDH*, whereas U6 snRNA served as the reference for miR-148a-3p. The stability of *GAPDH* and U6 was assessed by comparing their raw quantification cycle (Cq) values across the control, 2.5 µM, and 5 µM treatment groups in both cell lines. Relative expression was calculated using the $2^{-\Delta \Delta Cq}$ method [20,21].

### 2.6. Western Blot Analysis

Total protein was extracted from A549 and H460 cells in radioimmunoprecipitation assay (RIPA) lysis buffer (Thermo Fisher Scientific, Waltham, MA, USA) containing protease and phosphatase inhibitor cocktail (Thermo Fisher Scientific, Waltham, MA, USA). Protein content was quantified using the bicinchoninic acid (BCA) assay kit (Thermo Fisher Scientific, Waltham, MA, USA). Equal amounts of protein (40 µg per sample) were resolved on 10% SDS–PAGE gels alongside a molecular-weight standard and subsequently electrotransferred onto 0.45-µm nitrocellulose membranes (Thermo Fisher Scientific, Waltham, MA, USA). After blocking with 5% skim milk prepared in TBST for 1 h at room temperature, the membranes were incubated overnight at 4 °C with antibodies targeting DNMT1 (mouse monoclonal; Antibodies.com, Cambridge, UK; 1:1000), PTEN (rabbit monoclonal; FAGUS Antibody Services, Solihull, UK; 1:1000), and GAPDH (mouse monoclonal; Santa Cruz Biotechnology, Inc., Dallas, TX, USA; 1:1000), then incubated for 1 h at room temperature with the appropriate HRP-conjugated secondary antibodies: rabbit anti-mouse IgG (H+L) (Invitrogen, Thermo Fisher Scientific; 1:3000) for DNMT1 and GAPDH, and goat anti-rabbit IgG (H+L) (Invitrogen, Thermo Fisher Scientific; 1:3000) for PTEN. Protein signals were developed using an enhanced chemiluminescence substrate, and densitometric analysis was performed with ImageJ (version 1.54u7). The intensity of each target band was normalized to the corresponding GAPDH signal and multiplied by 100.

### 2.7. PTEN Promoter Methylation Analysis

Genomic DNA was isolated from vehicle-treated and 5-aza-dC-treated A549 and H460 cells using the DNeasy Blood & Tissue Kit (QIAGEN GmbH, Hilden, Germany). Bisulfite modification of the purified DNA was performed with the EpiJET Bisulfite Kit (Thermo Fisher Scientific, Germany). MethPrimer (https://www.methprimer.com/ (accessed on 1 June 2026)) was used to design *PTEN*-specific primer pairs that distinguish methylated from unmethylated bisulfite-converted DNA. Primer selection criteria included lengths of 18–25 nucleotides and approximate melting temperatures of 58–60 °C. Primer sequences are provided in Table 2. PCR products were resolved on 2% TBE agarose gels and detected by ethidium bromide staining. 

### 2.8. Statistical Analysis

Statistical analyses were performed using GraphPad Prism version 9.5 (GraphPad Software, San Diego, CA, USA). Each experiment was independently repeated three times, and data are presented as means ± standard deviations. Group differences were evaluated using one-way ANOVA followed by Tukey’s multiple-comparison test. All tests were two-sided, and *p* < 0.05 was considered statistically significant.

## 3. Results

### 3.1. Reduced PTEN and Increased DNMT1 Expression in LUAD and LUSC

**Figure 1:**
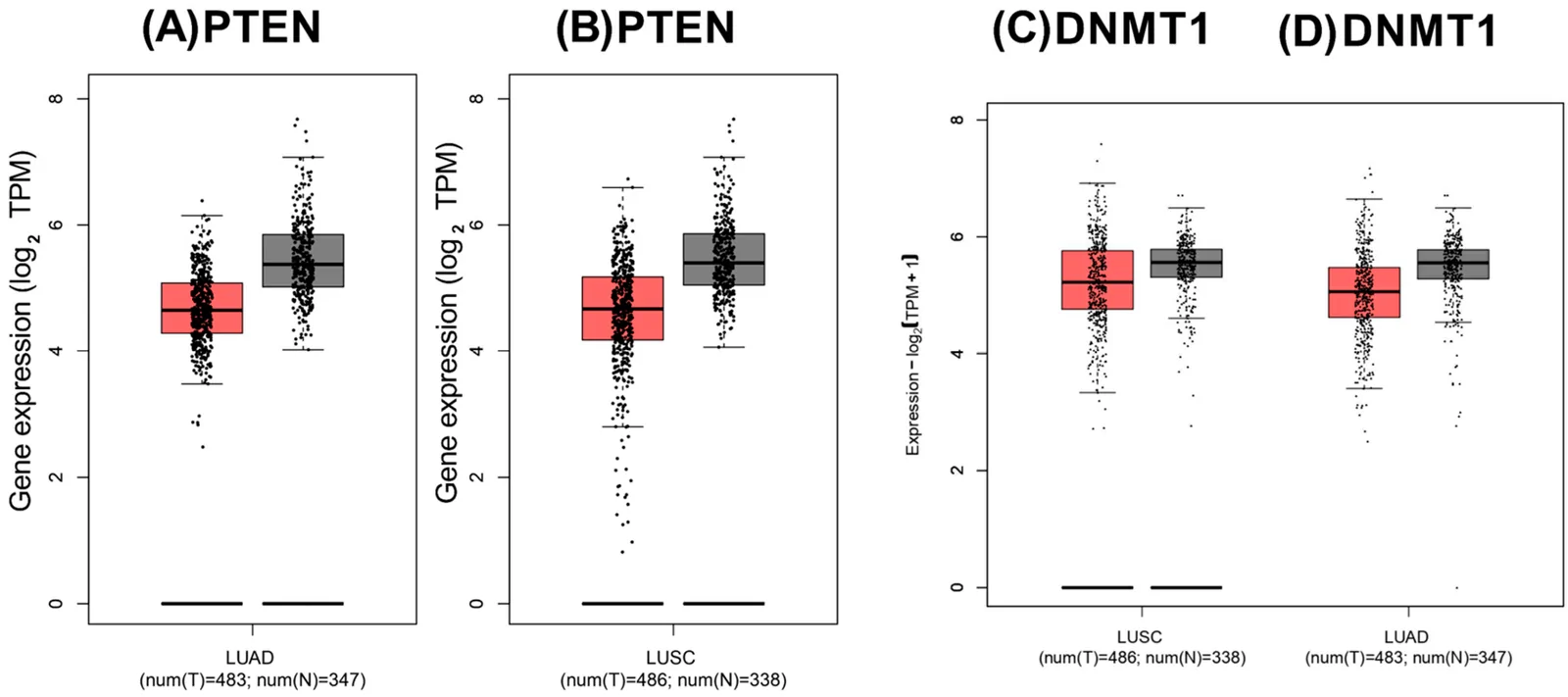
*PTEN* and *DNMT1* transcript abundance in LUAD and LUSC. GEPIA2 was used to compare PTEN transcript abundance in (**A**) LUAD and (**B**) LUSC and DNMT1 transcript abundance in (**C**) LUSC and (**D**) LUAD. The analyses included 483 LUAD tumors and 347 combined TCGA–GTEx normal lung samples and 486 LUSC tumors and 338 combined TCGA–GTEx normal lung samples. Identical dataset and analysis settings were applied to both genes.

Analysis using GEPIA2 showed lower *PTEN* transcript abundance in LUAD and LUSC tissues than in the corresponding non-cancerous lung samples (Figure 1A,B). Conversely, *DNMT1* transcript abundance was elevated in both NSCLC subtypes relative to normal lung tissue (Figure 1C,D).

### 3.2. PTEN Methylation–Expression Relationships Vary Across NSCLC Subtypes

**Figure 2:**
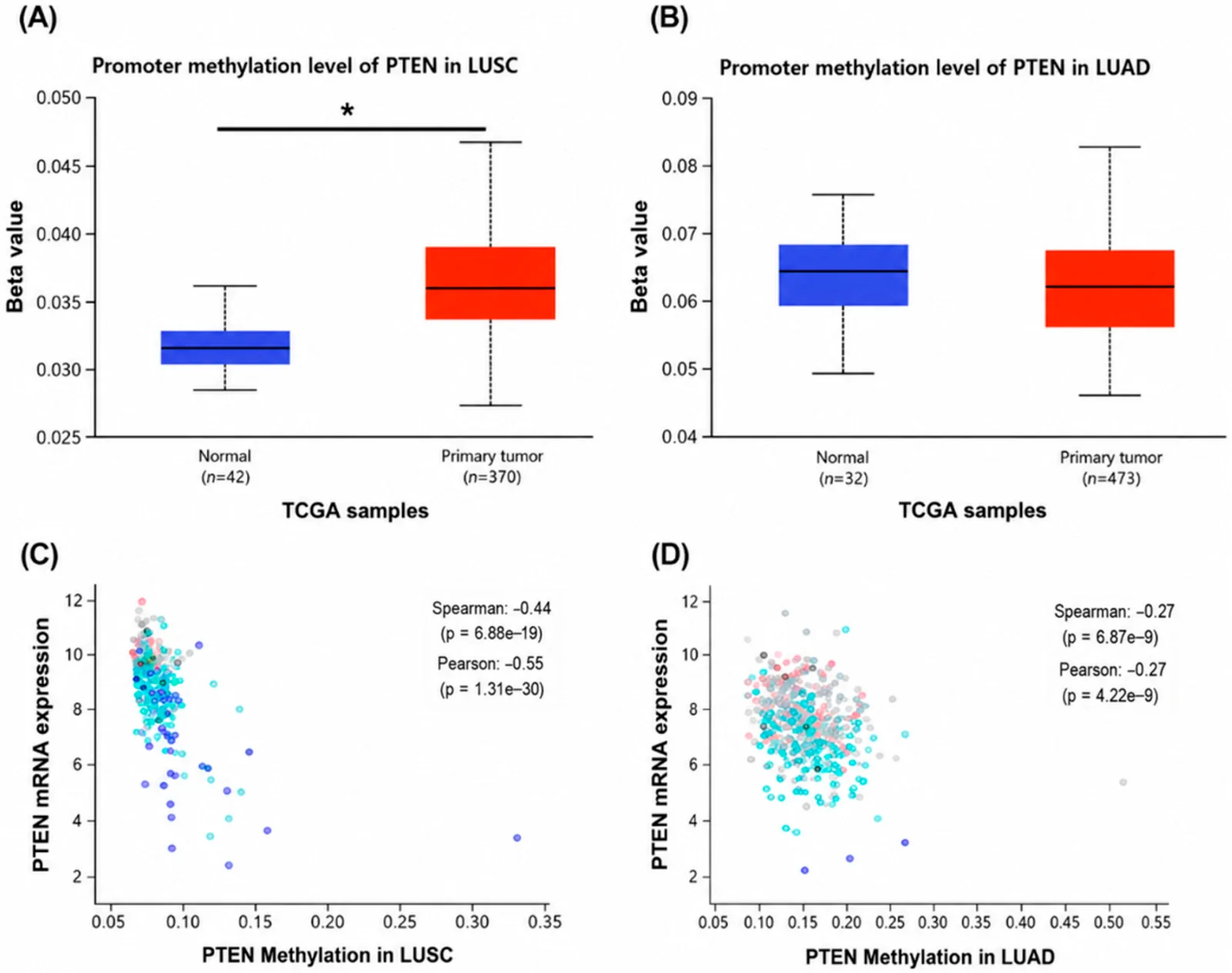
*PTEN* promoter methylation level and its association with transcript abundance in NSCLC cohorts. UALCAN was employed to compare the methylation level of the *PTEN* promoter between tumor and normal tissues in (**A**) LUSC and (**B**) LUAD. The association of *PTEN* methylation with its transcript abundance was assessed using cBioPortal in (**C**) LUSC and (**D**) LUAD. Spearman and Pearson correlation coefficients and corresponding *p* values are presented in the plots. In panel (**A**), the asterisk (*) indicates a statistically significant difference between normal and primary tumor samples (*p* < 0.05).

UALCAN analysis revealed higher *PTEN* promoter methylation in LUSC tumors than in normal lung tissues (tumor, *n* = 370; normal, *n* = 42; Figure 2A). In contrast, *PTEN* promoter methylation did not differ significantly between LUAD tumors and normal samples (tumor, *n* = 473; normal, *n* = 32; Figure 2B). Analysis using cBioPortal (version 7.0.0) showed an inverse association between PTEN methylation and transcript abundance in both tumor subtypes. The relationship was moderate in LUSC (Spearman ρ = −0.44, p = 6.81 × 10^−19^; Figure 2C) and weaker in LUAD (Spearman ρ = −0.27, p = 6.45 × 10^−9^; Figure 2D).

### 3.3. miRNet Identifies miR-148a-3p-Associated Gene Interactions

**Figure 3:**
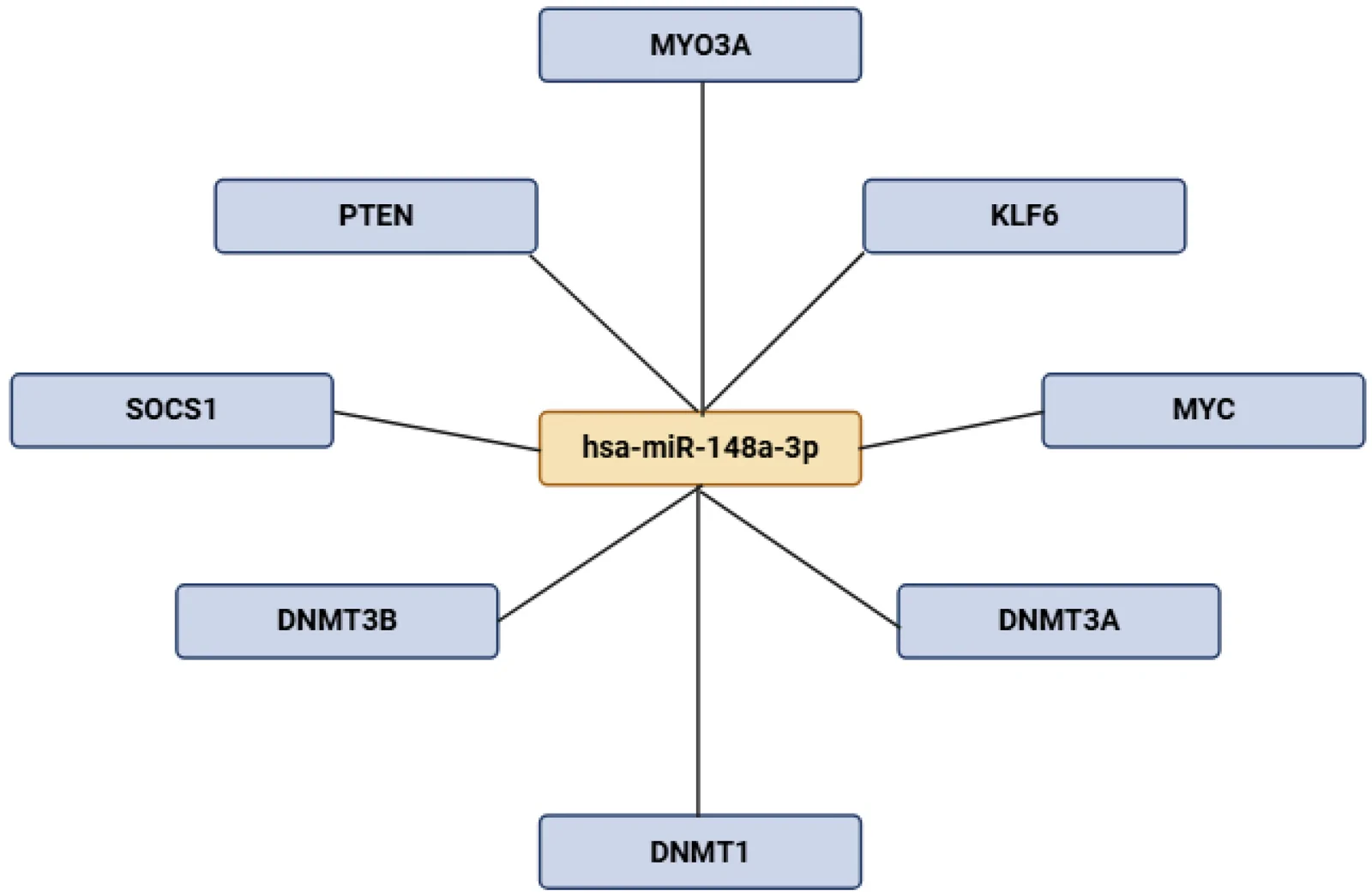
miR-148a-3p-associated gene network generated using miRNet. The network shows predicted or database-supported interactions between miR-148a-3p and selected target genes, including *PTEN* and *DNMT1*. The network was visualized using Cytoscape. Computationally identified associations require experimental validation.

The miRNet-derived network identified predicted or database-supported associations between miR-148a-3p and several genes, including *PTEN, DNMT1, DNMT3A, DNMT3B, MYC, KLF6, MYO3A*, and *SOCS1* (Figure 3). These computational associations do not establish direct regulatory interactions. 

### 3.4. 5-aza-dC Reduces MTT Metabolic Activity in A549 and H460 Cells

**Figure 4:**
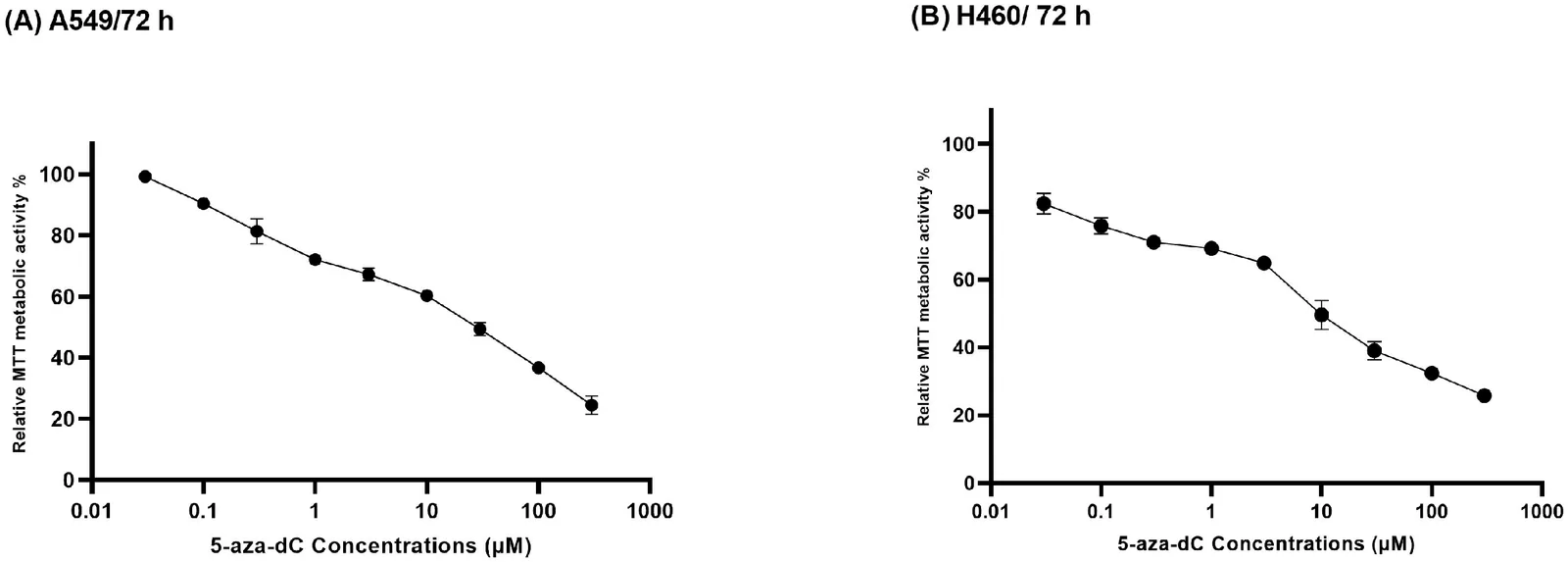
Concentration–response effects of 5-aza-dC on the metabolic activity of NSCLC cells. Relative MTT metabolic activity was measured in (**A**) A549 and (**B**) H460 cells after exposure to 0.03–300 µM 5-aza-dC for 72 h. The calculated IC_50_ values were 5.091 ± 0.81 µM for A549 cells and 5.068 ± 0.90 µM for H460 cells.

Exposure to increasing concentrations of 5-aza-dC produced a concentration-dependent reduction in relative MTT metabolic activity in both A549 and H460 cells (Figure 4). In A549 cells, metabolic activity declined from approximately 100% at the lowest tested concentration to approximately 25% at 300 µM. A comparable concentration-dependent response was observed in H460 cells, with metabolic activity decreasing to approximately 25% at the highest concentration. Four-parameter nonlinear regression analysis yielded IC_50_ values of 5.091 ± 0.81 µM for A549 cells and 5.068 ± 0.90 µM for H460 cells.

### 3.5. 5-aza-dC Induces Cell-Line-Dependent Alterations in PTEN, DNMT1, and miR-148a-3p Expression

**Figure 5:**
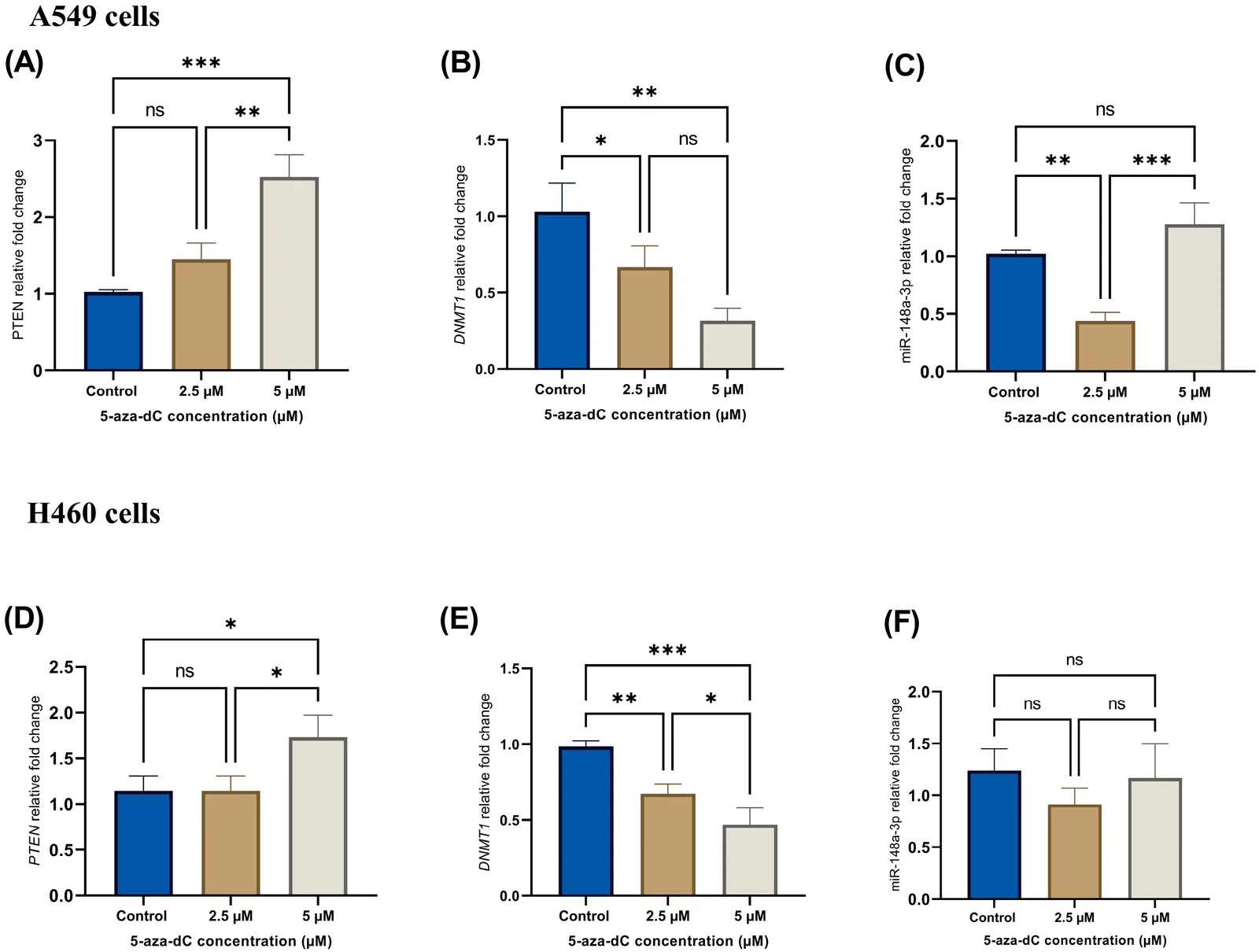
5-aza-dC-mediated modulation of *PTEN*, *DNMT1*, and miR-148a-3p expression in NSCLC cells. A549 cells were analyzed for (**A**) *PTEN*, (**B**) *DNMT1*, and (**C**) miR-148a-3p expression after treatment with 5-aza-dC at concentrations of 2.5 and 5 µM for 72 h. The corresponding analyses in H460 cells are shown for (**D**) *PTEN*, (**E**) *DNMT1*, and (**F**) miR-148a-3p. Transcript levels were determined by RT-qPCR and expressed relative to the vehicle-treated control. Data are presented as mean ± SD from three independent biological experiments. Group differences were evaluated using one-way ANOVA followed by Tukey’s multiple-comparison test. * *p* < 0.05, ** *p* < 0.01, *** *p* < 0.001; ns, not significant.

RT-qPCR revealed distinct concentration- and cell-dependent expression patterns after 72 h of 5-aza-dC exposure (Figure 5). In A549 cells, treatment with 2.5 µM 5-aza-dC produced a modest, non-significant increase in *PTEN* mRNA relative to the control. At 5 µM, PTEN expression increased further and was significantly greater than in both the control group (*p* < 0.001) and the 2.5 µM treatment group (*p* < 0.01; Figure 5A). *DNMT1* expression decreased at 2.5 µM (*p* < 0.05 versus the control) and showed a greater reduction at 5 µM (*p* < 0.01 versus the control). However, the difference between the two treatment concentrations was not significant (Figure 5B). A biphasic pattern was observed for miR-148a-3p in A549 cells. Its expression decreased at 2.5 µM relative to the control (*p* < 0.01) and then increased at 5 µM relative to the 2.5 µM group (*p* < 0.001). Importantly, miR-148a-3p expression at 5 µM did not differ significantly from the control (Figure 5C). In H460 cells, *PTEN* expression was unchanged at 2.5 µM but increased significantly at 5 µM compared with both the control and the 2.5 µM group (*p* < 0.05; Figure 5D). *DNMT1* expression decreased at 2.5 µM (*p* < 0.01 versus the control) and declined further at 5 µM (*p* < 0.001 versus the control; *p* < 0.05 versus 2.5 µM; Figure 5E). miR-148a-3p expression did not differ significantly among the H460 treatment groups (Figure 5F).

### 3.6. 5-aza-dC Enhances PTEN Protein Expression and Suppresses DNMT1 Protein Levels

**Figure 6:**
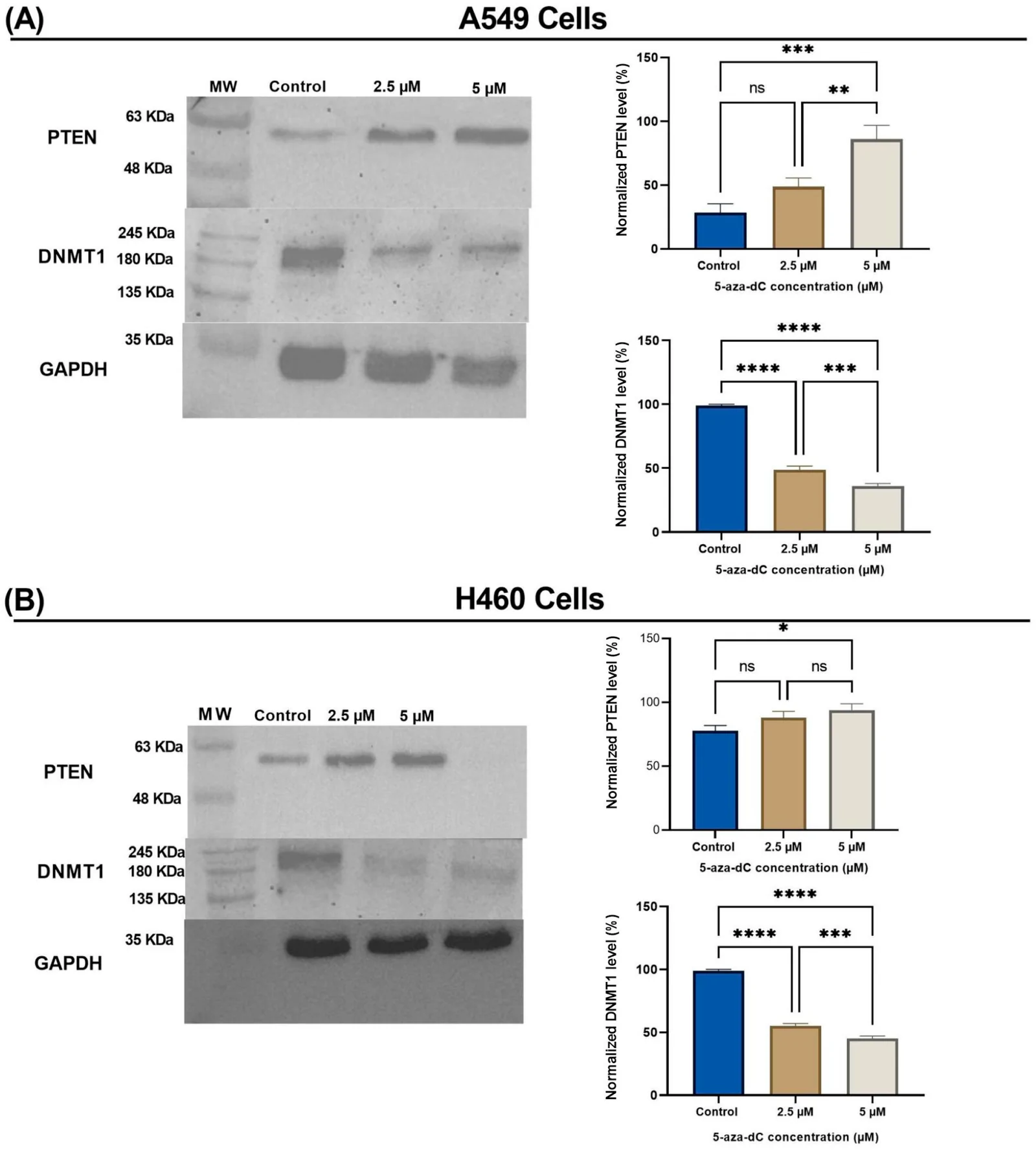
5-aza-dC-induced changes in PTEN and DNMT1 protein expression. Representative Western blots and corresponding densitometric analyses are shown for (**A**) A549 and (**B**) H460 cells treated with 2.5 and 5 µM 5-aza-dC for 72 h. PTEN, DNMT1, and GAPDH were detected on separate membranes containing the corresponding samples. For presentation, the lanes are displayed in the common order of control, 2.5 µM, and 5 µM. PTEN and DNMT1 band intensities were normalized to the corresponding GAPDH signals and expressed as percentages. The blots shown are representative of three independent biological experiments. Data are presented as mean ± SD. Group differences were evaluated using one-way ANOVA followed by Tukey’s multiple-comparison test. * *p* < 0.05, ** *p* < 0.01, *** *p* < 0.001, **** *p* < 0.0001; ns, not significant.

Western blotting with densitometric analysis revealed concentration- and cell-line-dependent alterations in PTEN and DNMT1 protein abundance (Figure 6). In A549 cells, PTEN protein abundance showed a non-significant increase at 2.5 µM but was significantly elevated at 5 µM compared with the control (*p* < 0.001) and the 2.5 µM group (*p* < 0.01). DNMT1 protein abundance was markedly reduced at both 2.5 and 5 µM compared with the control (*p* < 0.0001 for both comparisons), with an additional reduction at 5 µM relative to 2.5 µM (*p* < 0.001; Figure 6A). In H460 cells, PTEN protein abundance was not significantly altered by 2.5 µM treatment. Treatment with 5 µM produced a small but significant increase relative to the control (*p* < 0.05), while no significant difference was observed between the 2.5 and 5 µM groups. DNMT1 protein abundance decreased strongly at both treatment concentrations compared with the control (*p* < 0.0001) and was significantly lower at 5 µM than at 2.5 µM (*p* < 0.001; Figure 6B).

### 3.7. Qualitative Changes in PTEN Promoter Methylation Patterns Following 5-aza-dC Treatment

**Figure 7:**
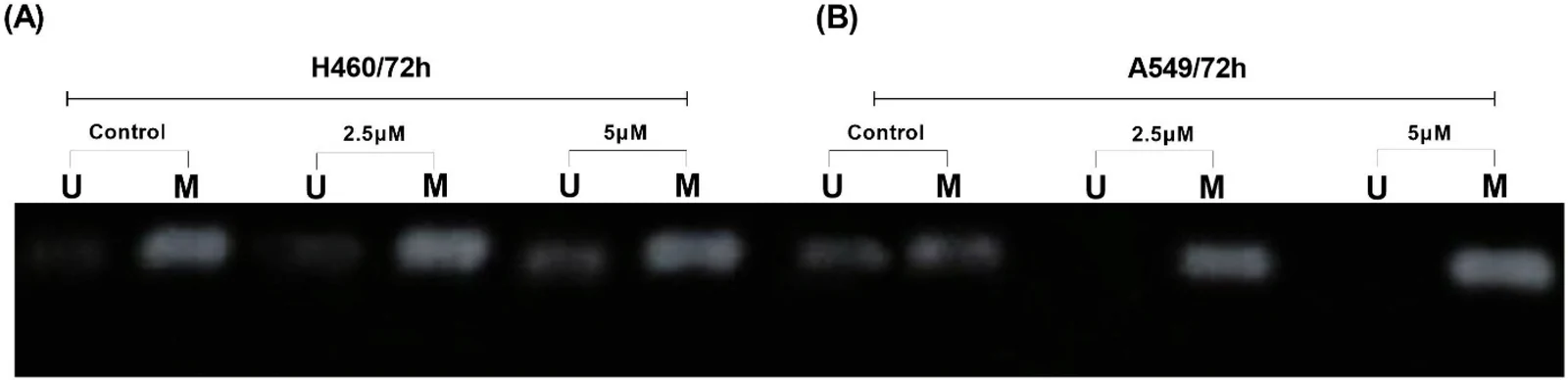
MSP-PCR assessment of *PTEN* promoter methylation following 5-aza-dC treatment. Methylated (M) and unmethylated (U) PTEN-specific amplification products were examined in (**A**) H460 and (**B**) A549 cells following treatment with 2.5 and 5 µM 5-aza-dC for 72 h. U, unmethylated-specific amplification; M, methylated-specific amplification.

MSP-PCR detected methylated *PTEN*-specific amplification in both A549 and H460 cells under control and treatment conditions (Figure 7). Weak unmethylated-specific amplification was also observed in selected H460 lanes; however, the methylated product remained detectable after treatment with 2.5 and 5 µM 5-aza-dC. In A549 cells, the methylated PTEN-specific amplification pattern remained prominent across all treatment groups. 

## 4. Discussion

5-aza-dC exposure increased *PTEN* expression and reduced *DNMT1* expression in A549 and H460 cells. Consistent with previous studies, bioinformatic analyses revealed reduced PTEN transcript abundance in both LUAD and LUSC and increased PTEN promoter methylation specifically in LUSC, together with elevated DNMT1 expression in both NSCLC subtypes, supporting a potential contribution of epigenetic dysregulation to NSCLC tumorigenesis [22,23]. Elevated *DNMT1* may help maintain methylation-dependent repression of tumor-suppressor loci [24,25].

Notably, 5-aza-dC treatment reduced *DNMT1* expression and increased *PTEN* expression, while MSP-PCR revealed persistent and heterogeneous methylation-specific amplification patterns. These findings are consistent with the established activity of 5-aza-dC, which promotes *DNMT1* depletion and subsequent DNA hypomethylation [26,27]. The observed increase in *PTEN* expression may therefore reflect changes in promoter methylation, together with indirect transcriptional or methylation-independent regulatory mechanisms. Similar findings have been reported in colorectal cancer cells, where 5-aza-dC-mediated *DNMT1* suppression was associated with *PTEN* re-expression, suggesting that this epigenetic relationship may extend across different tumor contexts [28].

Collectively, these results support an association between reduced *DNMT1* expression and increased *PTEN* expression following 5-aza-dC treatment in NSCLC cells.

Given that 5 µM approximated the 72-h MTT IC_50_ in both cell lines, the observed molecular changes may partly reflect cytotoxicity or selective survival of resistant cell populations. *PTEN* promoter hypermethylation was observed primarily in LUSC, whereas no significant difference was detected between LUAD and normal lung tissues. These findings suggest that promoter methylation may contribute substantially to *PTEN* silencing in LUSC, whereas additional mechanisms, including miRNA-mediated regulation and genetic alterations may also contribute to *PTEN* suppression in LUAD [29]. Because the experimental analysis included A549 and H460 cells but not a direct LUSC model, the cell-based findings should not be considered an experimental validation of the LUSC-specific methylation pattern.

A notable finding of the present study is the differential regulation of miR-148a-3p between A549 and H460 cells, highlighting a cell-type-specific epigenetic response. The contrasting miR-148a-3p responses may reflect differences in the genetic and epigenetic backgrounds of A549 and H460 cells [30,31]. A549 and H460 cells were selected to represent distinct histological and molecular backgrounds within NSCLC. A549 is a lung adenocarcinoma model, whereas H460 was derived from a large-cell lung carcinoma [32,33]. A549 harbors the *KRAS* G12S mutation, while H460 carries *KRAS* Q61H and *PIK3CA* E545K mutations [34]. These genomic differences, together with potential variation in basal methylation and miRNA regulation, may contribute to the cell-line-specific *PTEN* and miR-148a-3p responses to 5-aza-dC. However, this interpretation remains hypothetical, and the two models represent only a limited subset of the genomic and epigenomic heterogeneity of NSCLC [34,35].

Tumor-suppressive miRNAs have been shown to enhance the efficacy of targeted therapies by repressing oncogenic signaling pathways or restoring tumor suppressor function [36]. These multilayered regulatory networks underscore the complex interplay between epigenetic modifications and miRNA-mediated post-transcriptional regulation in shaping cancer cell behavior and drug sensitivity. Such complexity may partially explain the heterogeneous responses observed among NSCLC cell lines following treatment with epigenetic agents, including DNMT inhibitors.

Previous studies have reported direct regulation of DNMT1 by miR-148a/miR-148a-3p in several malignancies, including bladder, pancreatic, laryngeal, esophageal, and prostate cancers [37,38]. In contrast, the present study revealed a cell-specific response. In A549 cells, miR-148a-3p decreased at 2.5 µM and increased at 5 µM relative to the 2.5 µM group, although the 5 µM level was not significantly different from the control. No significant changes were detected in H460 cells. This variation may reflect differences in the basal epigenetic profiles of the cell lines, miRNA regulatory networks, or genetic backgrounds between NSCLC cell lines. It is also plausible that miR-148a-3p regulation is context-dependent and influenced by additional upstream epigenetic or transcriptional mechanisms. 

Further mechanistic validation is required through functional manipulation of miR-148a-3p and *DNMT1*, together with quantitative analysis of *PTEN* promoter methylation and assessment of PTEN-dependent signaling and cellular phenotypes.

## 5. Conclusions

In A549 and H460 cells, 5-aza-dC exposure coincided with higher *PTEN* expression and lower DNMT1 abundance, while MSP-PCR continued to detect heterogeneous *PTEN* methylation-specific products. miR-148a-3p responses were cell-dependent and did not support a definitive miR-148a-3p–*DNMT1–PTEN* regulatory pathway. The data support an association between 5-aza-dC exposure, reduced DNMT1 abundance, and increased PTEN expression, without establishing a causal DNMT1–PTEN regulatory association. Quantitative methylation assays and targeted functional experiments are needed to determine how *DNMT1*, miR-148a-3p, and *PTEN* are mechanistically connected in NSCLC.

## Figures and Tables

**Table 1:** Primer sequences used for RT-qPCR analysis.

Primer ID	Primer Sequence
*PTEN*	(Forward) 5′-TGAGTTCCCTCAGCCGTTACCT-3′
	(Reverse) 5′-GAGGTTTCCTCTGGTCCTGGTA-3′
*DNMT1*	(Forward) 5′-GAGGAAGCTGCTAAGGACTAGTTC-3′
	(Reverse) 5′-ACTCCACAATTTGATCACTAAATC-3′
*GAPDH*	(Forward) 5′-GTCTCCTCTGACTTCAACAGCG-3′
	(Reverse) 5′-ACCACCCTGTTGCTGTAGCCAA-3′
miR-148a-3p	(Forward) 5′-TGCGGTCAGTGCACTACAGAAC-3′
	(Reverse) 5′-CCAGTGCAGGGTCCGAGGT-3′
U6	(Forward) 5′-CTCGCTTCGGCAGCACA-3′
	(Reverse) 5′-AACGCTTCACGAATTTGCGT-3′

**Table 2:** Methylation-specific PCR (MSP-PCR) primers for *PTEN* promoter methylation analysis.

Primer ID	Primer Sequences	Amplicon Size
Methylated *PTEN*	(F) 5′-GGCGGCGGTCGCGGTTC-3′	71 bp
	(R) 5′-GACTCCCCGAAAACGCTAC-3′	
Unmethylated *PTEN*	(F) 5′-GAGAGATGGTGGTGGTTGT-3′	78 bp
	(R) 5′-AACTCCCCAAAAACACTACC-3′	

## Data Availability

The publicly available datasets analyzed in this study were accessed through GEPIA2, UALCAN, cBioPortal, and miRNet.

## Abbreviations

The following abbreviations are used in this manuscript:

5-aza-dC	5-aza-2′-deoxycytidine
AKT	Protein kinase B
ANOVA	Analysis of variance
ATCC	American Type Culture Collection
BCA	Bicinchoninic acid
cBioPortal	Cancer genomics data-analysis portal
cDNA	Complementary DNA
DMSO	Dimethyl sulfoxide
DNA	Deoxyribonucleic acid
DNMT1	DNA methyltransferase 1
FBS	Fetal bovine serum
GAPDH	Glyceraldehyde-3-phosphate dehydrogenase
GEPIA2	Gene Expression Profiling Interactive Analysis 2
HRP	Horseradish peroxidase
IC_50_	Half-maximal inhibitory concentration
LUAD	Lung adenocarcinoma
LUSC	Lung squamous cell carcinoma
miR-148a-3p	MicroRNA-148a-3p
miRNet	MicroRNA-centric network analysis platform
mRNA	Messenger RNA
MSP-PCR	Methylation-specific polymerase chain reaction
MTT	3-(4,5-dimethylthiazol-2-yl)-2,5-diphenyltetrazolium bromide
NSCLC	Non-small-cell lung cancer
PCR	Polymerase chain reaction
PI3K	Phosphoinositide 3-kinase
PTEN	Phosphatase and tensin homolog
RIPA	Radioimmunoprecipitation assay
RNA	Ribonucleic acid
RPMI-1640	Roswell Park Memorial Institute 1640 medium
RT-qPCR	Reverse-transcription quantitative polymerase chain reaction
SD	Standard deviation
SDS–PAGE	Sodium dodecyl sulfate–polyacrylamide gel electrophoresis
snRNA	Small nuclear RNA
TBE	Tris–borate–EDTA
TBST	Tris-buffered saline containing Tween 20
TCGA	The Cancer Genome Atlas
TSGs	Tumor-suppressor genes
U6 snRNA	U6 small nuclear RNA
UALCAN	Web-based cancer omics data-analysis portal
